# Windsock or Cobra Head Sign: A Distinctive Imaging Sign to Differentiate Type 1 Colonic Atresia From Hirschsprung's Disease

**DOI:** 10.7759/cureus.36786

**Published:** 2023-03-28

**Authors:** Richa Yadav, SSK Venkatesh, Dipin Sudhakaran, Vijay Mallayya Ganakumar

**Affiliations:** 1 Radiology, All India Institute of Medical Sciences, New Delhi, IND

**Keywords:** surgery, colonic atresia, barium enema, low intestinal obstruction, neonate

## Abstract

Colonic atresia is a rare cause of congenital low-type intestinal obstruction in the neonatal age group and may present as a surgical emergency if not diagnosed early. Clinically, it can pose a diagnostic dilemma for Hirschsprung disease, which involves a different treatment strategy. Therefore, an early and accurate diagnosis is paramount from a management and prognosis perspective. The contrast enema plays a crucial role in the diagnosis of the disease. The "Windsock or Cobra head sign" on the contrast enema, typically seen only in type 1 colonic atresia, can help radiologists and surgeons identify this disease. We report a case of a two-day-old neonate, including a clinical feature of low-grade intestinal obstruction with distinctive imaging signs of type 1 colonic atresia, which can help make a definitive diagnosis.

## Introduction

Most low-type intestinal obstructions are congenital in the neonatal age group and may present a surgical emergency. The incidence of neonatal intestinal obstruction is one in every 2000 live births [1]. Congenital colonic atresia is the rarest type of intestinal atresia, which requires early and accurate diagnosis for proper patient management to minimize the morbidity related to late diagnosis [2]. The etiology remains unclear, but the most accepted hypothesis is that the atresia is caused by an early prenatal vascular insult [3,4]. Imaging, especially a per-rectal contrast enema, plays an important role in diagnosing colonic atresia. Type 1 colonic atresia may mimic Hirschsprung's disease clinically, but if a contrast enema reveals a 'windsock or cobra head sign", it is characteristic of type 1 colon atresia. Our goal is to present the clinical and characteristic imaging findings and a brief overview regarding managing congenital type 1 colon atresia and emphasize the importance of this sign in the differential diagnosis of Hirschsprung's disease (HD).

## Case presentation

A two-day-old baby boy, born with normal-term vaginal delivery, was referred to pediatric emergency with complaints of progressive abdominal distention and absent passage of stool since birth. A detailed history revealed worsening symptoms after feeding. Birth history did not reveal any significance. Initially, her vitals were within the normal range, but then she developed mild respiratory distress and appeared mottled and dehydrated after episodes of vomiting. On examination, the abdomen appeared distended with visible peristalsis. The rectal examination revealed no meconium stain, but the anal opening arose at the usual site. The laboratory parameters appeared within normal limits.

**Figure 1:**
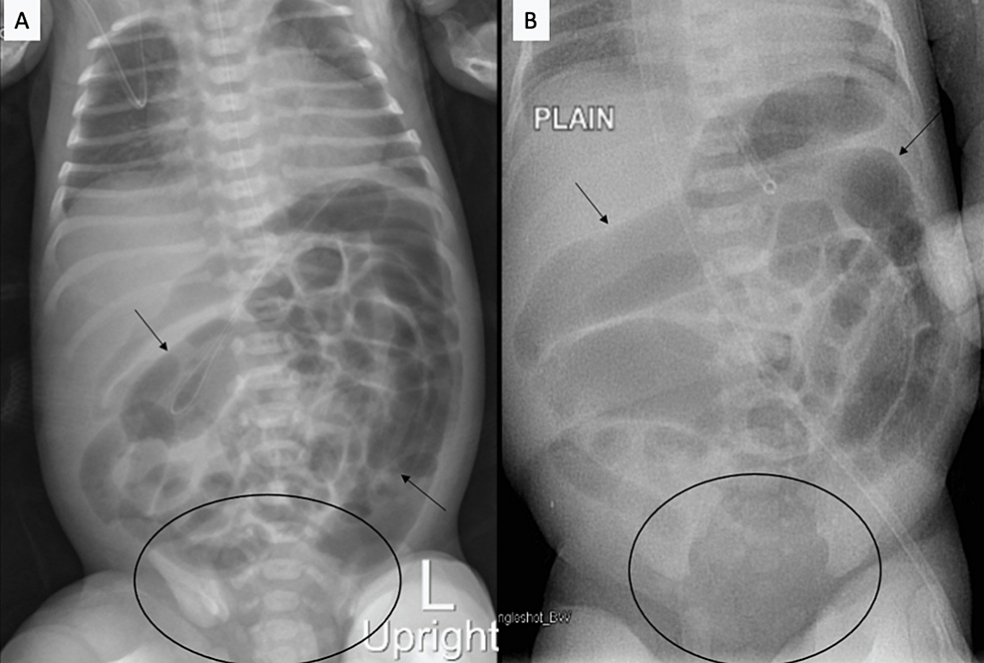
Abdominal radiographs of the neonate in upright (A) and supine (B) positions: showed gas-filled dilated bowel loops in the abdominal cavity (black arrow) with a paucity of rectal gas (black circle). No free peritoneal air or calcification was seen. These findings suggest the diagnosis of low-type intestinal obstruction with a differential of Hirschsprung disease at the top of the list.

A diagnosis of neonatal intestinal obstruction with a provisional diagnosis of Hirschsprung's disease (HD) was considered. The abdominal radiograph revealed dilated gas-filled bowel loops in the abdominal cavity with a paucity of gas shadow in the rectal region (Figures 1A, 1B), with no free intraperitoneal air or calcifications. 

**Figure 2:**
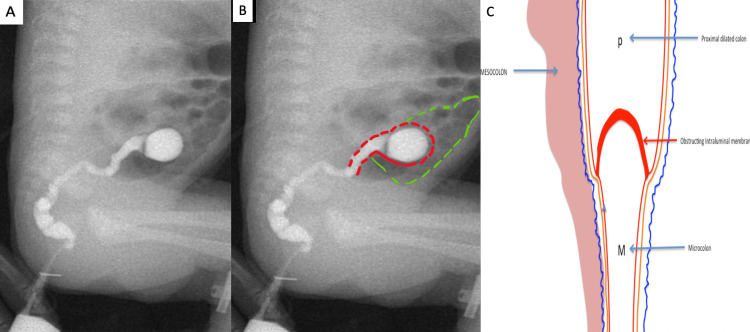
The contrast enema image in the left lateral (A, B) position shows a small caliber of the rectum and sigmoid (microcolon) in the proximal part with an abrupt ending into a rounded blind-ending sac (club shape or cobra head appearance [windsock sign, red dotted outline]). No retrograde flow of contrast is seen in the distal gas-filled dilated colon (green dotted outlines). Diagrammatic representation (C) of colonic atresia type1: shows the distal microcolon with an obstructing intraluminal membrane/diaphragm bulging into the dilated proximal colon, giving rise to the characteristic club shape/cobra head appearance. This characteristic sign is the "Windsock sign."

For further evaluation, rectal contrast enema performed under fluoroscopy guidance, and an appropriately diluted water-soluble contrast enema was injected rectally. It showed a small-caliber rectum and sigmoid colon (microcolon) with a preserved rectosigmoid ratio of 2:1. A round, contrast-filled, blind-ended pouch-like structure was depicted distally at the junction of the sigmoid along with a descending colon with no flow of contrast into the proximal colon (Figures 2A, 2B). This complete absence of retrograde flow of contrast into the proximal colon definitively ruled out the diagnosis of HD. Hence, a radiological diagnosis of type 1 colonic atresia with microcolon was made.

Outcome and follow-up

**Figure 3:**
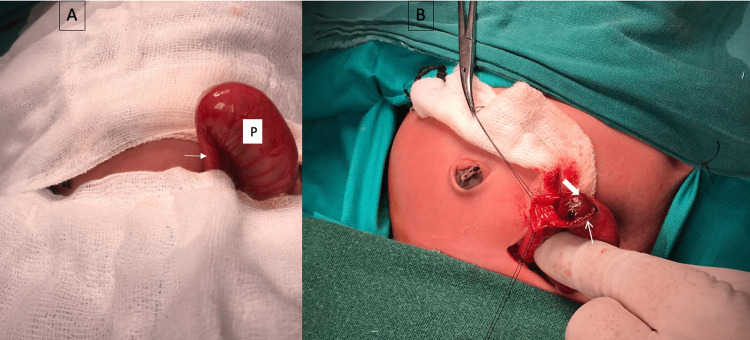
Intra-operative photographs (3A, 3B) showed a distal microcolon (arrow) with a dilated proximal colon (P). The photograph after the longitudinal incision at the distal segment (B) revealed the bulging membrane (thin white arrow) and the meconium at the tip of the opening in the membrane (thick white arrow).

Neonate underwent surgery due to worsening symptoms; imaging findings corroborated intraoperative findings. Open laparotomy revealed an intraluminal thin mucosal web in the sigmoid colon, bulging into an adjacent dilated descending colon, with the expulsion of meconium after the incision of the web. These findings confirmed the diagnosis of type 1 colonic atresia (Figures 3A, 3B). The histopathology of the resected specimen demonstrated the presence of ganglion cells, excluding the association of Hirschsprung's disease with colonic atresia in our case.

## Discussion

Congenital colonic atresia is among the rare causes of low-type intestinal obstruction, accounting for 1.8%-5% of all intestinal atresias, with an increased preponderance in males [1,2]. Unlike other intestinal obstructions, diagnosis is usually delayed because symptoms start a few days after feeding [2].

Several theories were proposed to explain the etiopathogenesis of colonic atresia (CA). The most accepted one was an early intrauterine vascular insult caused by extrinsic compression of bowel obstruction (internal hernia, volvulus) leading into aseptic necrosis and resorption [3,4]. The other causes included failure to recanalization of the intestinal epithelium, intestinal perforations, familial history, drugs (cocaine, amphetamine), and infection (varicella) [5-7]. Erskine et al. proposed that migration of emboli from the placenta might bypass the pulmonary circulation and reach the mesenteric vessels, thereby resulting in atresia of the colon [5-7].

An association between colonic atresia and HD is reported in 2% of patients [8]. Hence, a rectal biopsy must be included in initial surgical procedures to avoid the delayed diagnosis of associated Hirschsprung disease (HD) and its complications [8,9].

Clinically, newborns with colonic atresia generally present features of a low type of intestinal obstruction. Unlike another type of intestinal obstruction, the bauhin valve presents as a closed bowel loop obstruction. Hence, the onset of abdominal distention and vomiting is usually delayed [8,9].

Imaging plays a primary role in making a definitive diagnosis, excluding the close mimickers, and can be a guiding light for surgeons with regard to prognosis and management [10]. A plain abdominal radiograph is the first imaging investigation to start the evaluation of abdominal distention in a pediatric age group [10]. The findings are nonspecific for intestinal obstruction, which show gas-filled dilated bowel loops proximal to the atretic segment with a paucity of bowel gas in the rectal region (Figure 1). Free intraperitoneal air and calcification can be viewed as complications. Contrast enema is the subsequent investigation of choice in neonates for further approach [10]. Water-soluble contrast, preferably non-ionic water-soluble contrast with appropriate dilution, is injected per-rectally via an infant feeding tube (IFT). Foley catheters must be strictly avoided since the atretic segments are prone to rupture. The distinctive imaging features depend on the type of atresia described in Table 1.

**Table 1:** Illustrating the differentials and imaging characteristics of a low type of Intestinal obstruction in neonate.

Causes of Low Type Intestinal Obstruction	Clinical Manifestation	Radiographic Features	Contrast Enema
Hirschsprung Disease	Abdominal distention Unable to pass meconium within 48 hrs of birth.	Gas-filled dilated bowel loops in the abdomen with paucity of bowel shadow in the rectum.	Reversed recto-sigmoid ratio. Narrow caliber rectum with an abrupt transition point at the recto-sigmoid junction. Retrograde filling of contrast into the proximal colon.
Congenital Colonic atresia Type 1	Clinically, this is similar to the above. Clinical presentation is delayed, unlike intestinal atresia.	Same as above	Microcolon (unused small caliber rectum and sigmoid colon), Recto-sigmoid ratio-normal A contrast-filled “Cobra Head or Windsock” appearance at the transition point. The complete absence of retrograde filling of contrast into the proximal colon.
Small left colon syndrome Or Meconium Plug Syndrome	H/o is classical (Maternal DM). Clinical features are similar to those of functional rather than mechanical obstruction.	Similar	Microcolon with abrupt transition at the splenic flexure. Multiple filling defects represent meconium.
Meconium Ileus	Usually, manifestation of cystic fibrosis (20%).	Similar	Microcolon of the entire large bowel with impacted meconium in ileal loops.

**Figure 4:**
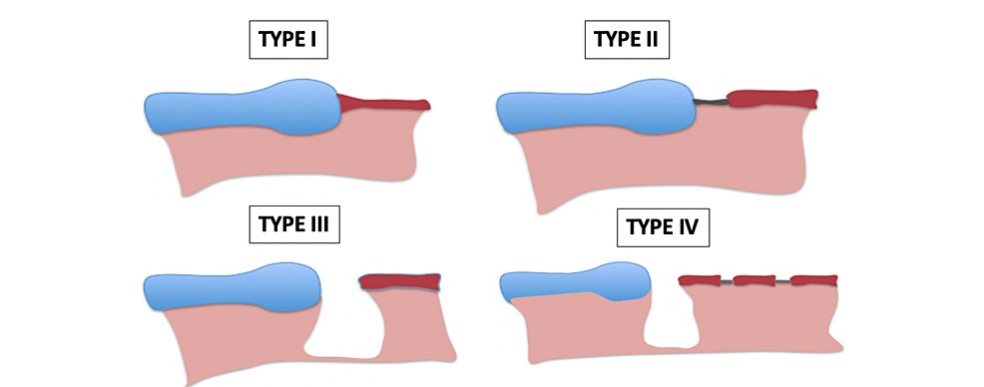
Grosfeld classification of colonic Atresia Type I: Intraluminal membrane (Windsock sign) or a very short atretic segment causing complete bowel obstruction, as in our case. Type II: Blind ending proximal bowel attached to distal microcolon by a fibrous cord along the edge of the mesocolon. There is no mesocolon defect. Type III: Similar to type II except that the connecting cord is absent and there is a V-shaped mesocolon defect that gives a ‘hook sign’ appearance. Type IV: Multiple segments of atresia, like a string of sausages; or a combination of types I and III.

Based on the revised classification of Martin et al. [10] and Grosfeld et al. [11], colonic atresia is classified into four types (Figure 4). Type 3 is the most common type of colonic atresia (Figure 4), while Type 1 is less frequent; the presence of an obstructing intraluminal membrane or a thin web characterizes that. In contrast, the enema study will appear as a microcolon (because it is unused) in the proximal part till the level of obstruction/web, with a characteristic round intraluminal contrast-filled sac bulging into the distal dilated gas-filled bowel along with a complete failure of retrograde flow. It provides the characteristics of the "Windsock Sign" or "Cobra Head Appearance" (Figure 2) [11]. This sign is characteristically observed only in type 1 of colonic atresia; hence, the other types and HD can be positively ruled out. It is similar to the sign observed in the intraluminal duodenal diverticulum. In the case of HD, there will be an abrupt transition and retrograde flow of contrast with the loss of the normal rectosigmoid ratio. Hence, the failure of complete retrograde contrast flow and a preserved rectosigmoid ratio of 2:1, along with the above characteristic sign, will exclude the diagnosis of HD [11,12]. The differentials of type 1 congenital colonic atresia are HD, the Ano-rectal malformation, etc. (Table 1). 

The management of colonic atresia includes early surgery to prevent morbidity and mortality. The repair included a primary or step-by-step anastomosis. The choice of surgical procedure depends on the location of the atresia. The preferred surgery for the proximal type of colonic atresia is resection and primary anastomosis. Colo-colic anastomosis with covering ileostomy is preferred in the distal or left-sided type of CA, as done in our case. Early perinatal diagnosis and individualized operation are the key steps in managing colonic atresia [9,12-14].

## Conclusions

Congenital colonic atresia is a rare form of low bowel obstruction in neonates with a slight male preponderance and may present a diagnostic dilemma clinically for Hirschsprung disease. Neonatal survival depends on early, accurate diagnosis and timely treatment. The contrast enema plays an important role in making the final diagnosis. The characteristic "windsock or cobra head sign" on the contrast enema can be a clue for radiologists and pediatric surgeons to make an early and accurate diagnosis of type 1 colon atresia. In 2% of the population, type 1 colon atresia is associated with HD; therefore, a rectal biopsy must be recommended before surgery in all cases.
